# High-Pressure Injection Injury of the Hand—A Rare but True Surgical Emergency

**DOI:** 10.3390/jcm14010072

**Published:** 2024-12-27

**Authors:** Mihaela Pertea, Stefana Luca, Malek Benamor, Mihai-Codrin Constantinescu, Andra-Irina Bulgaru-Iliescu, Alexandru Amarandei, Dan-Cristian Moraru, Khairi Saibi, Samar Ben Mrad, Alexandru Filip, Nina Filip

**Affiliations:** 1 Department Plastic Surgery and Reconstructive, Faculty of Medicine, “Grigore T. Popa” University of Medicine and Pharmacy, 700115 Iasi, Romania; mihaela.pertea@umfiasi.ro (M.P.); stefana.luca@d.umfiasi.ro (S.L.); constantinescu.mihai-codrin@d.umfiasi.ro (M.-C.C.); bulgaru-iliescu_andra-irina@d.umfiasi.ro (A.-I.B.-I.); alexamarandei@yahoo.com (A.A.); cristian-dan.moraru@umfiasi.ro (D.-C.M.); 2 Department of Plastic Surgery and Reconstructive Microsurgery, “Sf. Spiridon” Emergency County Hospital, 700111 Iasi, Romania; 3 Mohamed Kassab Institute of Orthopaedics, Plastic Surgery Department, El Manar University Tunis, Tunis 1068, Tunisia; khairisaibi@gmail.com (K.S.); samar.benmrad1@gmail.com (S.B.M.); 4 Department of Orthopaedics and Traumatology, Faculty of Medicine, “Grigore T. Popa” University of Medicine and Pharmacy, 700115 Iasi, Romania; alexandru-filip@umfiasi.ro; 5 Department of Orthopaedics and Traumatology, “Sf. Spiridon” Emergency County Hospital, 700111 Iasi, Romania; 6 Department of Morpho-Functional Sciences (II), Faculty of Medicine, “Grigore T. Popa” University of Medicine and Pharmacy, 700020 Iasi, Romania; nina.zamosteanu@umfiasi.ro

**Keywords:** high-pressure injection, hand, compartment syndrome, surgery, amputation

## Abstract

**Background/Objectives**: The aim is to bring attention to the existence of a rare type of trauma of the hand, high-pressure injection injury, that appears to be minor with negligible signs and symptoms within the first hours after the accident, but in reality, produces significant tissue destruction with severe consequences. Recognizing this type of trauma by medical personnel, understanding the mechanisms involved, and knowing the etiological and prognostic factors can lead to early treatment initiation and avoid severe mutilating sequelae. **Methods**: A retrospective study on 16 patients diagnosed with high-pressure injection injuries, including water, air, paint, paint mixed paint with thinner, petroleum jelly, and lime (washable paint containing calcium oxide). The patients’ epidemiological data, the time from accident to diagnosis, reasons for delayed diagnosis, treatments applied, and outcomes were recorded and evaluated. **Results**: All injuries occurred at the workplace due to negligence. Oil-based paint was implicated in 31.25% of cases. The most frequently affected anatomical region was the volar surface of the distal phalanx of the nondominant hand index finger. In one case, delayed presentation to medical care and diagnosis resulted in a compartment syndrome, requiring amputation. **Conclusions**: It is crucial to recognize and understand this type of trauma, as it constitutes an emergency due to its rapid progression. Delayed diagnosis can result in massive tissue destruction, potentially leading to the loss of limb segments and debilitating functional sequelae, which may severely impact a patient’s socio-professional life.

## 1. Introduction

High-pressure injection injuries of the hand are a rare, distinct, and potentially devastating form of trauma commonly encountered in industrial settings.

High-pressure injection trauma of the hand was first described in the English literature by Rees in 1937, who reported a case involving a mechanic who sustained a 4000-psi fuel oil injection injury while testing a diesel engine jet [1,2]. Such injuries occur when substances such as paint, grease, fuel, or hydraulic fluid are forcibly injected into soft tissues of the hand at high velocities, often exceeding 100 psi (7 bar). The most commonly affected patients are males working in industrial or domestic settings [3,4]. Injected substances may include air, water, oil-based paint, fuel oil, grease, petroleum jelly, veterinary vaccines, hydraulic fluid, solvents, plastics, and concrete [5].

The reported incidence is 1:600 hand injuries annually, with major hand surgery centers reporting between one and four cases per year [2]. The most frequent site of injection is the pulp of the index finger (50%) on the non-dominant hand (60–75% of reported cases in the literature), followed by the thumb and, less frequently, the little finger (10%) [2,6]. Since the entry point is typically a puncture wound with minimal initial symptoms, patients may delay seeking medical care, and the injury may be underestimated by medical personnel, leading to delayed diagnosis and treatment [7,8].

This type of injury involves multiple destructive mechanisms, resulting in severe tissue damage, and may necessitate amputation of the affected anatomical segment [5]. Prognostic factors include the chemical composition of the injected substance, the volume and temperature of the substance, the pressure at which it was injected, and the anatomical region affected [9,10,11]. Oil-based paint injections have a worse prognosis compared to water-based paint injections [3]. The least severe consequences are seen with water or air injections and veterinary vaccine injections [5]. Wong et al. propose a classification of this trauma in mild, medium, and severe categories, considering the etiological agent, the neurovascular condition of the affected anatomical segment, and the time interval from trauma to diagnosis and treatment initiation [12].

Early recognition of these injuries can help avoid the development of compartment syndrome, reduce the cytolytic effect of the injected substance, and lower the risk of infection, which is often polymicrobial [2,13]. For a better prognosis, high-pressure injection injuries must be diagnosed within the first 6 h and treated surgically [13,14]. Initial management should include assessing for potential systemic intoxication (evaluating the toxicity of the injected agent), tetanus vaccination, and the patient’s general biological status [15,16].

Surgical intervention performed under general or regional anesthesia typically involves incisions to decompress the affected anatomical segment and remove as much of the injected substance as possible, with thorough lavage of the infiltrated tissues [17]. Serial surgeries are often required, along with secondary wound closure, antibiotics, anti-inflammatory medication, and analgesics [18]. Amputation rates are reported to be between 30 and 48% [5]. Proper physical therapy significantly improves the chances of full recovery after high-pressure injection injuries [10].

We present a retrospective study of 16 patients diagnosed and treated for hand injuries caused by high-pressure injections of various substances. We evaluated the epidemiological characteristics of the study cohort, the etiological agents, and the context of the trauma. Additionally, we assessed the time intervals from the occurrence of the trauma to the establishment of the diagnosis, as well as the initiation of surgical or non-surgical treatment. Also, we evaluated the toxic potential of the injected substances, the time elapsed from the accident to presentation, diagnostic criteria, treatment methods, and functional outcomes.

## 2. Materials and Methods

The retrospective study included 16 patients who sustained high-pressure injection injuries localized to the hand. The patients were admitted and treated at the Plastic and Reconstructive Microsurgery Clinic at the “Saint Spiridon” Emergency Hospital in Iași between January 2014 and July 2023. Informed consent was obtained from all patients during the follow-up. The study received ethical approval from the ethics committee (approval number 113/21 November 2024).

Data were collected from patients’ observation sheets as well as from the hospital’s electronic system. The inclusion criteria for the study were patients over 18 years of age and a diagnosis of “high-pressure injection injury of the hand”. As this hospital does not treat patients under the age of 18, there are no additional exclusion criteria.

The following patient information was also collected: age, sex, occupation, comorbidities, the type of equipment involved, the injection site, the type of equipment involved in the injection (industrial or domestic), the nature of the trauma—voluntary/involuntary, the context domestic/work-related, the injected substance, the injection site, the time interval from the accident to presentation, the diagnosis and initiation of treatment, and complications such hematomas, seromas, skin ischemia or necrosis, infections, foreign body reactions, cysts, oleogranulomas, and fibrocystic tumors. Follow-up assessments were performed at 2, 4, and 9 months with the last evaluation occurring at one year.

Unfortunately, data regarding the pressure under which the substances were injected could not be recorded in any of the cases.

The diagnosis was established through a thorough medical history, consideration of the context and mechanism of the accident, and clinical examination, including local examination of the skin, vascularization (capillary refill), active and passive mobility of the affected segment, and sensitivity. Radiological examination was performed in all cases for radio-dense substances and, in a few cases, ultrasonography was also recommended. Delayed diagnosis and treatment were caused by the patients’ late presentation to medical care and the medical staff’s failure to recognize the injury’s severity. In cases requiring surgical intervention, the procedure was performed under locoregional anesthesia (axillary or infraclavicular block) with a tourniquet applied at the arm level, without exsanguination of the thoracic limb or any segment. Local anesthesia (digital block) was avoided to prevent exacerbation of edema and/or vasospasm. In cases requiring serial surgical interventions, locoregional anesthesia was used each time. In the case presenting lymphangitis extending from the affected finger to the middle third of the arm, the tourniquet was not applied.

Both local and systemic treatments received by patients during hospitalization were recorded. Functional outcomes (active and passive) and sensory assessments were performed using Two-Point Discrimination (2PD) and Semmes–Weinstein (SW) tests. Disabilities of the Arm, Shoulder, and Hand (DASH) score was calculated and patient satisfaction was evaluated using the Michigan Hand Outcomes Questionnaire (MHQ) scale, which includes six criteria: overall hand function, daily living activities, pain intensity, work activities, aesthetic appearance, and patient satisfaction.

## 3. Results

**Figure 1:**
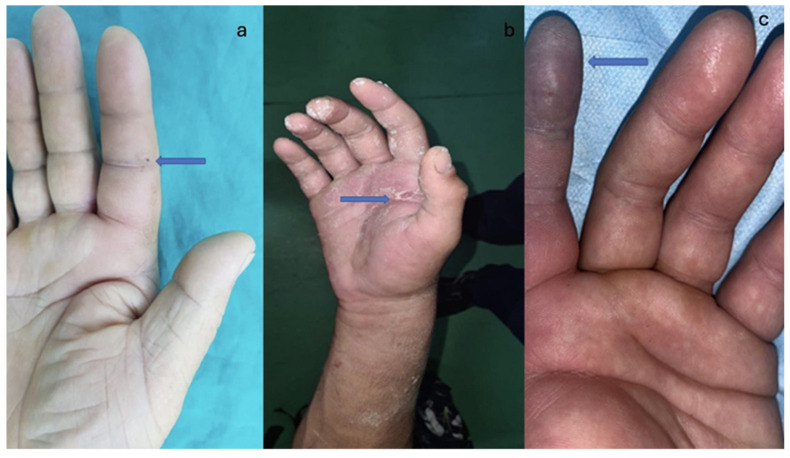
Punctiform entry wound. (**a**). Second phalanx of the index finger of the left hand. (**b**). Distal palmar flexion crease. (**c**). Distal phalanx of the index finger of the left hand.

All the patients in the studied group were male and aged between 19 and 63 years, with an average age of 39.1 years. Four patients were diagnosed with comorbidities such as hypertension. In these cases, they received appropriate antihypertensive treatment as prescribed and in accordance with the cardiologist’s recommendations. No other associated pathologies were recorded as the patients were relatively young, as reflected in the average age. All cases were involuntary accidents, occurring due to negligence at the workplace, resulting from failure to adhere to safety regulations; the remaining were domestic accidents. In all instances, the entry wound was punctiform, sometimes nearly imperceptible macroscopically (Figure 1).

The substances injected in the studied patient group were oil-based paint in five cases (31.25%), air in three cases (18.75%), petroleum jelly in two cases (12.5%), paint mixed with thinner in one case (6.25%), and lime (containing calcium oxide, with a high pH) in two cases (12.5%). Water was injected in three cases (18.75%).

**Figure 2:**
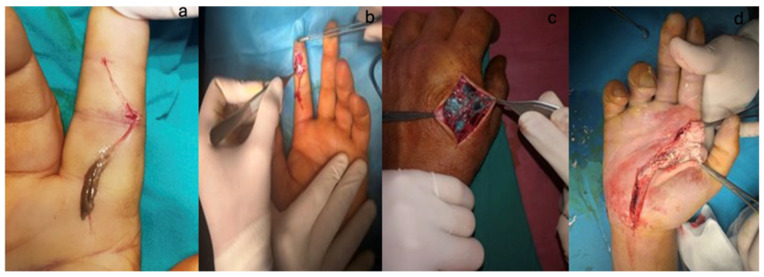
High-pressure injection. (**a**). Petroleum jelly. (**b**). Paint mixed with thinner. (**c**). Paint. (**d**). Lime.

The anatomical regions where the injection entry points were identified include the digital pulps in eight cases (50%), the palm in four cases (25%), the dorsal side of the hand in two cases (12.5%), and the second phalanx in two cases (12.5%). The most frequently affected area was the index finger in 10 cases, followed by the palm in 4 cases and the dorsal side of the hand in 2 cases. The left hand was affected in 11 cases (68.75%) (Table 1, Figure 2).

The time interval from the moment of the accident to the initiation of treatment ranged from 2 to 26 h, with an average of 8.62 h (Table 2). This delay in diagnosis and consequently in treatment initiation was due, in some cases, to the patients’ failure to seek timely medical care and in the remaining cases to the medical personnel’s failure to recognize the trauma and its severity.

In cases where the patient presented within the first 6 h after the accident, the clinical signs were mild pain and minimal edema. Late presentation to a physician was associated with severe pain, edema, sensory disturbances, and vascular impairment (delayed capillary refill), with some cases showing a discoloration of the skin, either pallor or cyanosis, and the formation of blisters, accompanied by compartment syndrome.

This clinical presentation was observed in cases where the injection site was at the level of the digital pulp. In 9 out of the 16 cases, diagnosis was made after the recommended 6-hour window. In the studied cases, the causes of these delays were attributed to the patients not presenting to medical care in a timely manner in six cases, while in the other three cases, the trauma was not recognized by medical personnel, and the patients were sent home with nonsteroidal anti-inflammatory treatment. In these cases, the symptoms worsened, and upon returning to the doctor, they were referred to a plastic surgeon for urgent treatment (Table 2).

The results indicate that in eight cases (50%), surgical treatment was not required, and conservative management sufficed. For the cases requiring surgical intervention, an average of 2 debridements per case was necessary: in two cases, three stages of debridement were performed, while in four cases, only two stages of surgical debridement were needed.

**Figure 3:**
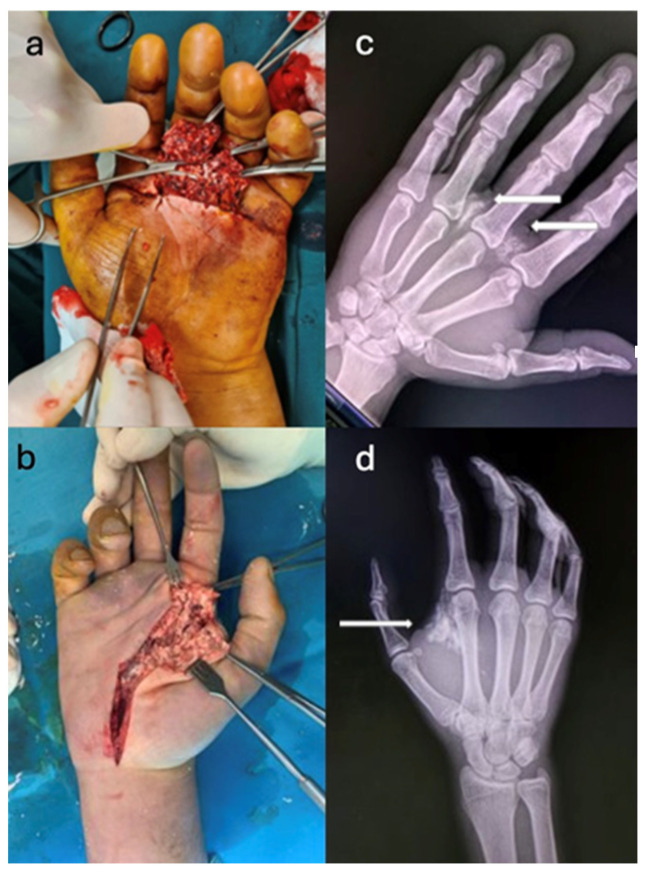
(**a**,**c**). High-pressure injection wounds with washable lime-based paint (calcium oxide) radio-opaque. (**b**,**d**). Radiological view.

The imaging investigations performed included anteroposterior and lateral radiographs, which detected the presence of injected substances when they were radio-opaque, as well as their diffusion into the surrounding tissues (Figure 3).

No signs of generalized intoxication or local or systemic allergic reactions to the injected substances were noted in any case.

**Figure 4:**
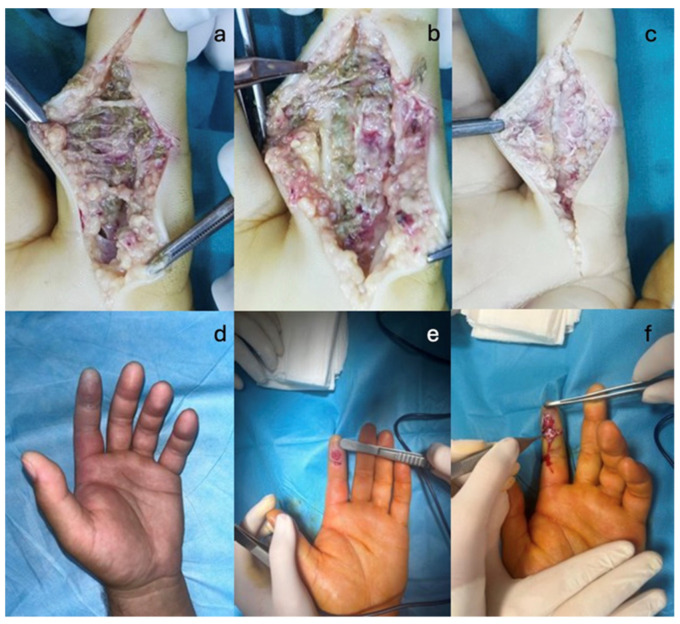
High-pressure injection wound: (**a**–**c**)—debridement and removal of petroleum jelly in 3 surgical stages; (**d**,**e**)—paint injection with compartment syndrome; (**f**)—finger incision with evacuation of the injected substance.

Surgical interventions were performed under loco-regional anesthesia (infraclavicular or axillary block). The surgical approach involved excision of the entry wound, followed by a wide incision extending into un-infiltrated tissue. In all cases, except those involving air or water as the injected substance, aggressive infiltration of all local and distant tissues was detected, not only along the vascular-nervous pedicles or tendon sheaths. This made the removal of the injected substance challenging, with no single surgical intervention being sufficient, always requiring serial debridement in 2 or even 3 stages, followed by secondary wound closure. Intraoperative lavage with chemical agents to remove the foreign substance was abundant, persistent, aggressive, and relatively difficult, as the substances were very adherent to the tissues (Figure 4).

**Figure 5:**
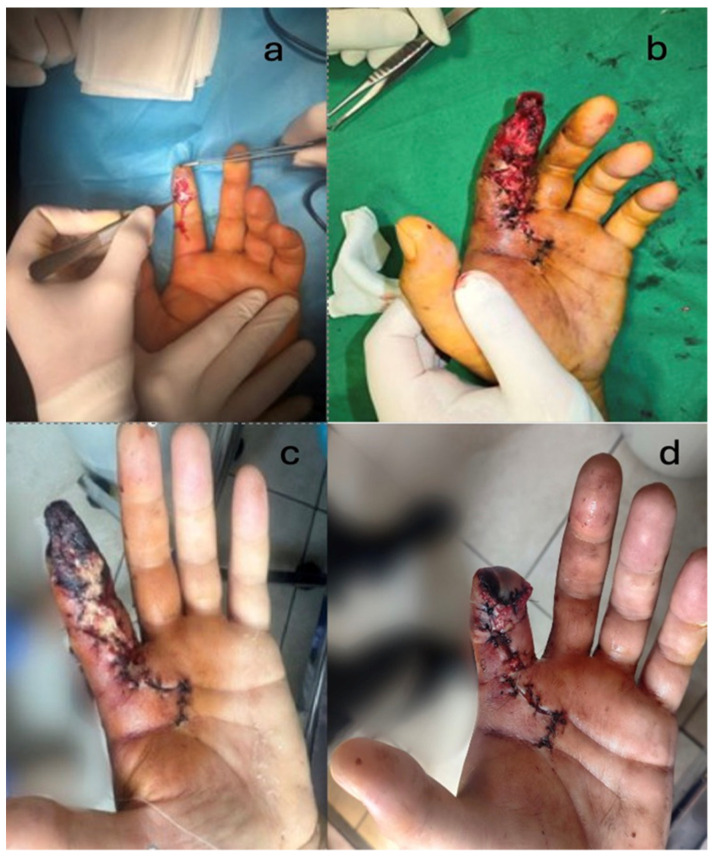
(**a**–**c**). Serial debridements. (**d**). Amputation of the second phalanx of the index finger. In this particular case, the necrotic lesions were extensive, requiring serial debridements. The etiology was attributed both to the delayed diagnosis, which led to the onset of compartment syndrome, and to the significantly higher combined toxicity of the two substances involved: paint, known to cause severe tissue damage, and solvent, which also has high toxicity.

In three cases from the studied group, where the injected substance was paint (in two cases) and paint mixed with thinner (in the third case), compartment syndrome was diagnosed upon admission. In this latter situation, in the first surgical intervention, the foreign material was evacuated and the compartment syndrome was treated simultaneously. However, within 2–3 days postoperatively, extensive pulp necrosis developed, despite the use of anticoagulant and antiplatelet therapies. Repeated removals of necrotic tissue were performed, but in the end, it necessitated the amputation of the necrotic segment: in this case, at the level of the distal interphalangeal joint (DIP) (Figure 5).

Throughout hospitalization/treatment, patients received prophylactic antibiotic therapy and vasodilator treatment and were kept at constant temperatures to avoid additional vasospasm. Nonsteroidal anti-inflammatory treatment was administered, and in severe cases with significant edema or compartment syndrome, dexamethasone was given 1–2 times per day. Pain management therapy was provided throughout the hospital stay, and physical therapy began during hospitalization. Acute renal failure or toxic shock was not diagnosed in any of the cases. No complications such as postoperative hematomas or seromas, skin ischemia or necrosis, infections, foreign body reactions, cysts, oleogranulomas, or fibrocystic tumors were recorded.

**Figure 6:**
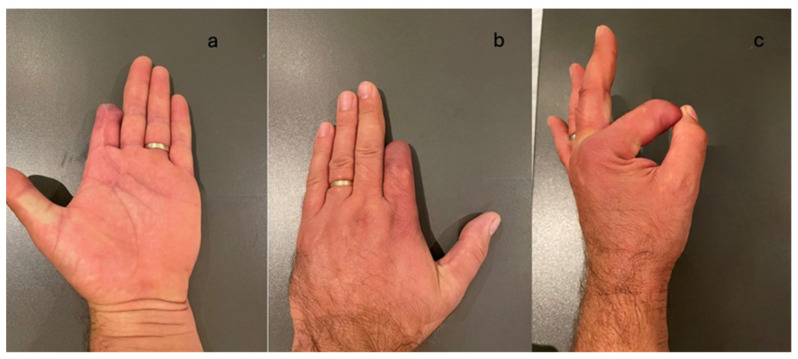
After the 3-month post-operator view. (**a**) Volar aspect. (**b**). Dorsal aspect. (**c**). Major functional sequelae.

According to the outcome evaluation scores, 15 out of 16 cases had good results, with no major motor or sensory functional deficits noted. In the case requiring an amputation of the middle phalanx of the second digit (D2), a functional deficit was documented in the hand (reduced grip strength, cylindrical grasp, thumb-amputated stump pinch on D2) along with an aesthetic deficit, resulting in low patient satisfaction and even frustration regarding the missed diagnosis during the first visit to the regional hospital. This delayed referral to a specialized hand surgery center, thereby favoring the development of compartment syndrome (Figure 6).

## 4. Discussion

A high-pressure injection injury is an extremely severe trauma that may lead to the need for amputation of a limb or limb segment. Often, due to minimal symptoms and a barely visible entry wound, this type of trauma is initially treated superficially or even unrecognized, with the severity of such an injury remaining unknown [19,20]. The amputation rate is influenced by the time elapsed from the moment of the accident until the diagnosis and subsequent treatment are established; for instance, the literature reports an increase in amputation rates in these cases from 58% to 88% [11]. Stark notes that patients show a much better prognosis when the injury is treated within a maximum of 10 h post-trauma [21].

In the studied group, the average time to initiate treatment was 8.62 h, indicating a relatively long delay, suggesting that this condition is still relatively underrecognized. The amputation rate is also influenced by the etiologic agent, with oil-based paint resulting in amputation rates of up to 80% of cases due to its high cytotoxicity, which triggers catastrophic inflammatory reactions [22,23,24]. This has a direct impact on tissues, leading to ischemia and necrosis. Thus, both the time from injury and the etiologic agent are critical prognostic factors. In our study group, of the six patients with paint injection, only one (16.66%) developed distal finger necrosis, resulting in distal phalange amputation. In this case, the injected substance was a mixture of paint and solvent, with toxicity heightened by the combination of these two chemicals. These findings align with reports in the literature [3,25,26,27].

Another factor influencing prognosis is the pressure at which the substance enters the skin. Naturally, lower injection pressures tend to cause less extensive injuries [3]. As mentioned, a limitation of the current study is the inability to record the pressures under which the substance was injected. Regarding the volume of the injected substance, a large volume, particularly if injected under high pressure, can cause significant tissue damage, including the potential development of compartment syndrome [22]. High-pressure injection into an “expandable” tissue (e.g., the palm) has a better prognosis than when it occurs in a small non-expandable space (e.g., a finger).

The literature on this pathology consists mainly of case reports or reviews in the literature. This study includes a cohort of 16 patients, making it one of the largest studied groups [5]. The characteristics of our retrospective study align with the literature findings: all cases involved male patients (100%), primarily affecting the index finger of the non-dominant hand (Wieder reports over 50% D2 involvement [10]), typically resulting from domestic or occupational accidents involving industrial substances. Notably, this study includes two cases involving washable paint, a substance rarely documented in this type of pathology. Hogan’s review (1966–2003) included a number of 435 high-pressure injection injuries recording a mean average patient age of 34.7 years, while in the present study, this mean age was 39.12 years [5]. In our cases, all surgical interventions were performed under locoregional anesthesia, with local anesthesia contraindicated. Exsanguination of the affected limb segment was also not recommended, with only tourniquet placement and usage advised. The average number of surgical debridements in the current study was two. In contrast, the literature review reported by Hogan et al. in 2006 did not record the average number of debridements required for high-pressure injection injuries [5]. In cases with significant edema and generalized lymphangitis affecting the entire upper limb, as seen in one case in our cohort, we consider that the simple use of a tourniquet may not be advisable. Infection is possible in such injuries, though this was not observed in our cohort, as cultures collected during the initial and serial interventions were negative.

The literature reports the potential for delayed complications such as squamous cell carcinomas as a late consequence [6]. All studies, including this one, underscore the severity of this type of trauma, its possible under-recognition or misjudgment, and its onset with minimal clinical signs and often imperceptible traumatic marks, which frequently lead to delayed recognition, diagnosis, and treatment—resulting in significant disabilities for affected patients [28].

The limitations of the study are given by the relatively small group of patients, but this is contextual to the rarity of this type of hand trauma. Also, correlations could not be made between the pressure of the injected substance and the extent of the tissue destruction because the pressure at which the different substances were injected could not be known and recorded.

## 5. Conclusions

High-pressure injection injuries are rare and seemingly “benign” but with a severe course and potential for significant functional and aesthetic sequelae that may lead to disability. This type of pathology must be known and well understood, not only by hand and plastic surgeons but also by emergency physicians and general practitioners, who are often the first to encounter these patients at the trauma site or shortly afterward. An early diagnosis and timely treatment will always improve the prognosis of such injuries, though the outcome is also influenced by several uncontrollable factors (substance type, volume, injection site, and pressure).

## Figures and Tables

**Table 1:** Distribution of the cohort by age, sex, entry wound location, and injected substance.

Nr.	Age	Sex	Entry Point Location	Injected Substance
1	26 y	M	P3F2 LH	air
2	44 y	M	P2F2 LH	petroleum jelly
3	32 y	M	P3F2 LH	paint with thinner
4	56 y	M	P3F2 LH	paint
5	47 y	M	dorsal side LH	paint
6	63 y	M	Palm LH	air
7	50 y	M	P3F2 LH	water
8	19 y	M	Palm LH	lime
9	29 y	M	P3F3 LH	paint
10	30 y	M	P3F3 RH	petroleum jelly
11	32 y	M	Palm RH	lime
12	54 y	M	P1F3 LH	paint
13	21 y	M	dorsal side LH	paint
14	59 y	M	P2F2 RH	water
15	39 y	M	Palm LH	water
16	25 y	M	P3F2 RH	air

y = year, M—male, P—phalanx, H—hand, R—right, L—left.

**Table 2:** Clinical and radiological signs, treatment, and results in the studied group.

	Time from Accident	Clinical Signs	Rx Exam	Treatment	Results
1	1 h	Punctiform wound, minimally visible, mild pain, moderate edema	-	conservative	No motor or sensory functional impairment
2	10 h	Punctiform wound, significant edema, intense pain	-	surgical (3 stages of surgery), anti-inflammatory, prophylactic antibiotic, anticoagulant	No functional sequelae after physiotherapy
3	22 h	Intense pain, compartment syndrome, cyanosis of P3, Lymphangitis of the hand, wrist, forearm, and arm	-	incision, lavage, serial debridements (3 stages of surgery), necessary amputation of P2F2, antibiotic, anti-inflammatory	Functional amputation stump
4	3 h	Moderate pain and edema Normal capillary refill	+	conservative treatment anti-inflammatory	No functional se-quelae after physiotherapy
5	8 h	Intense pain, intense edema, delayed capillary refill	-	surgery—2 steps antibiotic, anti-inflammatory anticoagulant	No functional sequelae after physiotherapy
6	15 h	Intense pain, intense edema, delayed capillary refill	-	conservative treatment anti-inflamatory anticoagulant	No functional sequelae
7	2 h	No symptoms, punctiform wound	-	conservative treatment	No functional sequelae
8	17 h	Moderate pain, intense edema	+	surgery—2 steps, antibiotic, anti-inflammatory, anticoagulant	No functional sequelae after physiotherapy
9	16 h	Intense pain, intense edema	-	surgery—2 steps, antibiotic, anti-inflammatory, anticoagulant	No functional sequelae after physiotherapy
10	4 h	No symptoms, punctiform wound	-	Conservative treatment	No functional sequelae
11	10 h	Pain, edema,	+	Surgery—1 step antibiotic, anti-inflammatory, anticoagulant	No functional sequelae after physiotherapy
12	5 h	No symptoms, punctiform wound	-	Surgery—1 step antibiotic, anti-inflammatory, anticoagulant	No functional sequelae
13	7 h	Pain, edema, moderate lymphangitis	-	surgery—2 steps, antibiotic, anti-inflammatory, anticoagulant	No functional sequelae after physiotherapy
14	6 h	Pain, edema		Conservative treatment	No functional sequelae
15	4 h	No symptoms, punctiform wound	-	Conservative treatment	No functional sequelae
16	8 h	Pain, edema, moderate lymphangitis	-	Conservative treatment	No functional sequelae

Rx—radiological exam, h—hour, P—phalanx, F—finger.

## Data Availability

The original contributions presented in the study are included in the manuscript; further inquiries can be directed to the corresponding authors. The data can be obtained from the corresponding author upon request. The original contributions presented in this study are included in the article.
